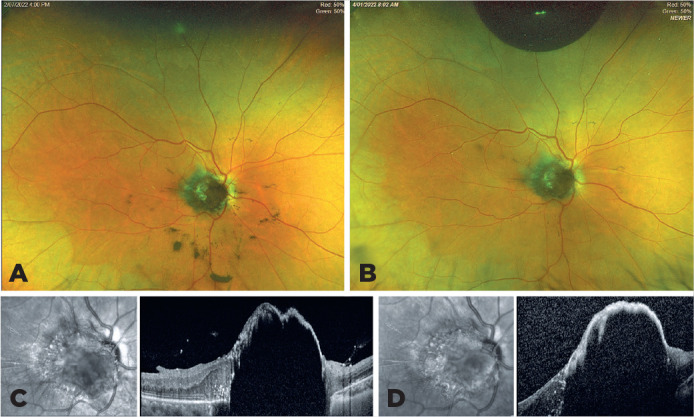# Features and management of a case of melanocytoma with vitreous seeding

**DOI:** 10.5935/0004-2749.2024-0306

**Published:** 2025-02-11

**Authors:** Carlos Augusto Ferraresi Sampaio, Gustavo Rosa Gameiro, Carolina Correa Valente, Zelia Maria Correa

**Affiliations:** 1 Escola de Medicina, Pontifícia Universidade Católica do Paraná, Londrina, PR, Brazil; 2 Departamento de Oftalmologia e Ciências Visuais, Escola Paulista de Medicina, Universidade Federal de São Paulo, São Paulo, SP, Brazil; 3 Centro Universitário Lusíadas, Santos, SP, Brazil; 4 Bascom Palmer Eye Institute, Miller School of Medicine, University of Miami, Miami, FL, USA; 5 Sylvester Comprehensive Cancer Center, Miller School of Medicine, University of Miami, Miami, FL, USA

A patient presented with a dark-brown tumor and melanotic vitreous cells in the amblyopic right eye (OD) (Figure A). OCT revealed a sonolucent optic disc tumor (Figure B). Differentials for this finding included metastatic cutaneous melanoma, necrotic uveal melanoma, and melanocytoma with vitreous seeding^(1^,^2)^. A vitrectomy confirmed the diagnosis of a melanocytoma with low metastatic risk (Class 1, PRAME-negative) and an excellent outcome^(1^,^3)^ (Figures C and D). Optic disc melanocytomas occasionally enlarge, causing visual loss due to spontaneous tumor necrosis or compressive neuropathy^(1^,^4)^. Malignant transformation occurs in 1%-2% of the lesions^(5)^.

**Figure:**